# Age-Related Dynamics of Lung-Resident Memory CD8^+^ T Cells in the Age of COVID-19

**DOI:** 10.3389/fimmu.2021.636118

**Published:** 2021-03-29

**Authors:** Nick P. Goplen, In Su Cheon, Jie Sun

**Affiliations:** ^1^Division of Pulmonary and Critical Medicine, Department of Medicine, Mayo Clinic, Rochester, MN, United States; ^2^The Robert and Arlene Kogod Center on Aging, Mayo Clinic, Rochester, MN, United States; ^3^Department of Immunology, Mayo Clinic, Rochester, MN, United States

**Keywords:** viral pneumonia, influenza, resident memory, pathology, homeostasis, age

## Abstract

Following respiratory viral infections or local immunizations, lung resident-memory T cells (T_RM_) of the CD8 lineage provide protection against the same pathogen or related pathogens with cross-reactive T cell epitopes. Yet, it is now clear that, if homeostatic controls are lost following viral pneumonia, CD8 T_RM_ cells can mediate pulmonary pathology. We recently showed that the aging process can result in loss of homeostatic controls on CD8 T_RM_ cells in the respiratory tract. This may be germane to treatment modalities in both influenza and coronavirus disease 2019 (COVID-19) patients, particularly, the portion that present with symptoms linked to long-lasting lung dysfunction. Here, we review the developmental cues and functionalities of CD8 T_RM_ cells in viral pneumonia models with a particular focus on their capacity to mediate heterogeneous responses of immunity and pathology depending on immune status.

## Introduction

“Infectious diseases are no respecters of wealth, power, or personal merit. Pandemic infectious disease is one situation where we cannot accept Margaret Thatcher's view [there is no such thing as society]. With a fast spreading respiratory virus, for example, everyone is ultimately in the same boat” (Peter C. Doherty concluding remarks in *Pandemics*, 2013). Respiratory viruses that infect the lower airways such as influenza virus and severe acute respiratory syndrome coronavirus 2 (SARS-CoV2) can cause severe acute lung injury (ALI) and are serious public health challenges. A year after the initial outbreak, SARS-CoV2 infection has resulted in more than 95 million cases and 2 million deaths globally (https://coronavirus.jhu.edu). Conventional T cells, particularly CD8 cytotoxic T cells, play important roles in the control of respiratory viral infection (1, 2). Additionally, CD8 T cells can form a long-lived immunological memory that protects from reinfection of the same or related viruses (3). Among the different subsets of memory CD8 T cells, tissue-resident memory T cells (T_RM_) that reside within the respiratory tract provide superior immunity against viral re-infections (4). Therefore, vaccines that can elicit robust CD8 T_RM_ cells are highly promising for the prevention/amelioration of future pandemics. Conversely, recent studies have suggested that exaggerated CD8 T_RM_ cell presence and/or uncontrolled CD8 T_RM_ cell function could lead to chronic pathogenic sequelae in the lungs (5, 6). Here, we will review recent literature on pulmonary CD8 T_RM_ cell development and maintenance and discuss their roles in immune protection as opposed to how they may provoke pulmonary pathologies when not tightly regulated. We primarily use influenza virus infection studies as the model for this review.

### Pulmonary Memories Fade Away

Pulmonary CD8 T_RM_ cells poised for rapid responsiveness, contribute substantially to immune protection of the host against previously encountered viral pathogens (4). As in other organs, pulmonary T_RM_ cell function appears to be dependent on *in situ* proliferation and the production of IFN-γ which activates the vasculature enabling recruitment of innate and adaptive responses (4, 7–10). Compared to T effector (T_EM_), T central (T_CM_), and T peripheral (T_PM_) memory cells that collectively circulate through blood, lymph, peripheral and secondary lymphoid organs, T_RM_ cells are transcriptionally and functionally distinct (11–16). The lung is one of few sites where CD8 T_RM_ cells are relatively short-lived and not permanently lodged in tissues compared to the limited number of organs investigated (17–19). Their loss over time has been attributed to migration from the parenchyma to the airways where they encounter a hostile environment eventually leading to their apoptosis (19). Additionally, pulmonary T_RM_ cells can re-enter the circulation and migrate to the draining lymph nodes where they re-establish residency, contributing to their loss from lung tissue (18). Of note, lung T_RM_ cell loss can be mitigated by local prime-boost strategies and/or repeated antigen exposure (20). Given the potential for their short life-span and their importance in clearing subsequent respiratory viral infections, it is critical to understand the environmental and immune-status cues that regulate T_RM_ cell differentiation, maintenance, and function in the lung in order to exploit their benefits through immunotherapies such as vaccines.

### Pulmonary T_RM_ Cells—the Human Experience

Counterparts to T_RM_ cells discovered in mice exist in all organs investigated in humans (11, 21). The lung faces constant microbial exposure, yet histology snapshots suggest the distal airways are remarkably sterile environments in the absence of acute infection. Accordingly, *in situ* estimates suggest human lung explants contain as many as 10 billion memory T cells (22). There is a diverse antigen-specific CD4 and CD8 T cell presence in most lungs including up to 10% of T cells that respond to influenza virus challenge with proliferation (22). Like CD8 T cells, CD4 T cells in the human lung appear transcriptionally primed for response (23, 24). While the resident CD4:CD8 memory T cell ratios vary by compartment (airway vs. parenchyma), 20–50% of pulmonary CD8 T cells expected to be critical for anti-viral memory responses, display a recently activated phenotype indicated by HLA-DR antigen on their surface (22, 25, 26), suggesting active vigilance.

Tracking of donor lung T cells following pulmonary transplantation, indicates T_RM_ cells are found sparsely in the blood at any given time, similar to what is observed in mouse studies (6, 26, 27). Further, donor and recipient airway T_RM_ cell transcriptional profiles overlap indicating a shared signature imparted by the lung microenvironment despite disparate HLA matches (26). As in mouse studies, a substantial fraction of human lung CD8 T_RM_ cells express multiple inhibitory receptors, suggesting a strong stimulus may be needed for their re-activation (24). Relative to peripheral blood memory T cells, human CD69^+^ pulmonary CD8 T_RM_ cells almost universally express CD29, CD49a, CXCR6, and PSGL-1 with heterogenous expression of CD103 and CD101. Despite this heterogeneity, strong stimulation through the T Cell Receptor (TCR) results in proliferation of the majority of human T_RM_ cells with their progeny exhibiting enhanced polyfunctional capacity relative to their parents (28). This suggests T_RM_ cells act as sentinels in human lung mucosa and are important for maintaining sterility of alveolar spaces.

### What Makes a Pulmonary T_RM_ a Pulmonary T_RM_?

Recent barcode lineage-tracing and single-cell transcriptome analyses found that a subset of T cell clones possesses a heightened capacity to form T_RM_ cells, as enriched expression of T_RM_-fate-associated genes is already apparent in circulating effector T cell clones (13). Consistently, following initial trafficking to the lung, T_RM_-like phenotypes are observed as early as 2 weeks following influenza infection and these phenotypes, but not numbers, are stable in the airways, lung parenchyma, and trachea for up to 3 months (17, 29). Pulmonary T_RM_ cells have been defined inconsistently throughout the literature, as warranting caution when comparing studies.

While pulmonary CD8 T_RM_ cell definition(s), differentiation, maintenance, and functions have largely been established from monoclonal T cell receptor (TCR) transgenic models, polyclonal experiments give a more heterogeneous and physiological relevant picture of T_RM_ cells coexisting within the same tissue, but have not been widely reviewed. Markers (e.g., CD69, CD103, CD49a, CXCR6, and PD-1) typically used to identify pulmonary CD8 T_RM_ cells in mice are heterogeneously co-expressed within T_RM_ populations (5, 6, 27, 29–32). For example, E-cadherin in the lung is expressed in the cell-cell junctions between bronchiole epithelium (33). Although E-cadherin-binding CD103 is intrinsically important for cytotoxic capacity (34) and is expressed on nearly 100% of T_RM_ in the skin, CD103 is heterogeneously expressed in lung T_RM_ cells, inhibits T_RM_ cell motility, and is not required for heterosubtypic protection against influenza. Conversely, although the collagen IV-binding integrin CD49a is a less common marker used for the identification than CD103, it is required for the heterosubtypic immunity against influenza infection (28, 29).

Furthermore, CD103 is expressed at a substantially lower frequency on the T_RM_ cells that form the bulk of the protective response vs. influenza nucleoprotein (D^b^-NP_366−374_) in C57BL/6 mice compared to another immune-dominant epitope from viral polymerase peptide (D^b^-PA_224−233_) (5). Nonetheless, parabiosis studies indicate both of these phenotypically different populations exhibit similar degrees of tissue residency 2 months following infection (6). Though the significance is unclear, this immunodominant population (responding to D^b^-NP_366−374_) in a secondary response that mostly lacks CD103 expression, abundantly expresses classic exhaustion markers (PD-1, TIM-3, LAG-3, and TIGIT) relative to D^b^-PA_224−233_ and K^b^-OVA_SIINFEKL_ –specific T_RM_ and memory CD8 T cells in the circulation (5, 6). These insights from various studies highlight the marked epitope-specific CD8 T_RM_ cell heterogeneity within the pool of polyclonal T_RM_ cells directed against the same pathogen. Indeed, data from organ donors indicates a diverse TCR repertoire against influenza virus, suggesting that heterogeneity is quintessential in the local pulmonary response (28).

### Cellular and Molecular Networks Involved in the Control of Pulmonary CD8 T_RM_ Cell Density

**Figure 1:**
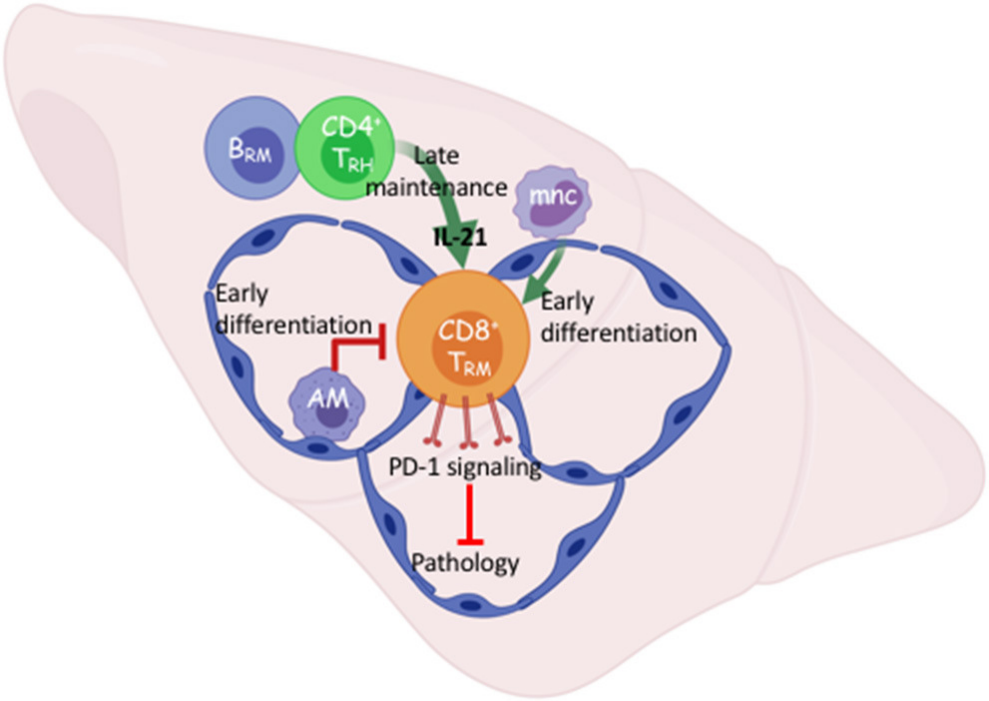
Early and late cellular networks involved in Trm cell differentiation and maintenance. After viral respiratory pneumonia, early pulmonary CD8 T_RM_ cell differentiation is driven by re-encounter with antigen presented *via* interstitial classic monocytes (mnc) and opposed by alveolar macrophages that maintain lung homeostasis. B cell-dependent tissue-resident CD4 helper T cells (T_RH_) support T_RM_ cell maintenance through IL-21 dependent survival. T_RM_ intrinsic PD-1 signaling prevents pathology in the absence of infectious virus. Created with BioRender.

It is becoming clearer that local immune interactions influence CD8 T_RM_ cell numbers without affecting the circulating memory pool. Alveolar macrophages (AMs) are a self-renewing population of airway-resident cells seeded early in embryonic development (35). AMs maintain lung homeostasis and respond to inflammatory cues. Absence or dysfunction of AMs in severe influenza infection leads to exacerbated pulmonary pathology and enhanced mortality (36, 37). In studies where we were investigating the effects of PPAR-γ in the macrophage compartment on influenza severity, intrinsic absence increased the density of pulmonary T_RM_ cells and long-term stromal disrepair indicated by persistent inflammation and collagen deposition (38, 39). We subsequently found that depletion of AMs prior to influenza infection, but not during the CD8 T cell contraction phase, enhanced T_RM_ cell density without affecting the circulatory memory compartment (Figure 1) (38). This suggests AMs have an early influence on the lung microenvironment that governs *in situ* T_RM_ cell differentiation. It is not currently clear what subtype of CD169^+^ AMs are responsible for limiting the T_RM_ cell compartment nor by what means. Conversely, bone-marrow derived monocytes trafficking to the site of infection enhance the early antigen-presentation required for T_RM_ cell differentiation in the lung (40). Yet, inflammatory macrophages in the gut mediate heterogeneous T_RM_ cell differentiation by contributing to the pro-inflammatory milieu (41).

In contrast to the limiting of the T_RM_ cell compartment by innate resident macrophages, we and others have recently shown that a population of CD4 tissue-resident helper T (T_RH_) cells aid the persistence of pulmonary CD8 T_RM_ cells following influenza infection (42, 43). This novel population of T_RH_ cells simultaneously exhibits T follicular helper (T_FH_)-like properties that enhance the local B cell response and tissue-resident memory T cell features. CD4 T_RH_ cells are the major cellular sources of IL-21 in the tissue, and blockade of IL-21 signaling at the memory stage diminished CD8 T_RM_ cell survival specifically in the D^b^-NP_366−374_ population.

While the influenza response in the lung is not an active chronic infection, viral RNA remnants may cause persistent pathology (44). In persistent viral infection in the brain, provision of IL-21 by T follicular-like tissue-resident CD4 T cells likely promotes ATP production in local CD8 T cells through enhancing electron transport chain efficiency (45). Our data suggests this could be a means by which local CD8 T cells differentiate and persist in response to IL-21. Nonetheless, a local interaction between CD8 and CD4 T cells is required for optimal T_RM_ cell responses following both acute and persistent viral infections (Figure 1). Importantly, this cellular network was responsible for local secondary protection against heterologous infection mediated by the influenza-specific CD8 T_RM_ cells. Interestingly, T_RH_ cell development requires the presence of B cells (43); thus there exists a local interplay among adaptive immune cells for the maintenance of pulmonary lymphocyte memory following viral pneumonia. Understanding how the local cellular networks modulate immune protection may aid the development of mucosal vaccines. Additionally, understanding the molecular cues governing their persistence will likely be important to elicit proper T_RM_ cell responses through immunotherapies.

Unlike the majority of inflamed organs investigated, where it merely enhances T_RM_ cell differentiation, local antigen signals are required for the establishment of pulmonary CD8 T_RM_ cell (17, 46). As briefly mentioned above, T_RM_ cells with TCRs of different specificities against influenza epitopes, exhibit different phenotypes and have distinct requirements for their maintenance (5). At the transcriptional level, polyclonal CD8 T_RM_ cells also vary in their programs between T_RM_ cells of different specificities (5, 6). The TCR is likely playing an active role in these differences. Just as the quality of TCR signals can determine CD8 T cell fate in the circulation, lower affinity TCR signals enhance the potential to differentiate into pulmonary T_RM_ cells (47–49).

Furthermore, the duration and amount of antigenic signals seem important for establishing the diversity of the T_RM_ cell pool against a given respiratory pathogen. For instance, the differential persistence of influenza NP vs. PA antigen at the memory phase clearly dictates the distinct phenotypes of the T_RM_ cells against the two antigens (5). Influenza virion contains many more NP molecules than PA molecules and NP proteins and/or NP_366−374_ peptide-MHC-I complex are present for a longer period and potentially in a much higher amount than PA proteins or PA peptide-MHC-I complex at the memory phase (50). In agreement, influenza NP-specific (D^b^-NP_366−374_), but not PA-specific (D^b^-PA_224−233_), T_RM_ cells receive chronic TCR signaling at the memory phase, leading to the development of an “exhausted-like” phenotype (characterized by the high expression of co-inhibitory molecules including PD-1 and Tim-3) in D^b^-NP_366−374_ T_RM_ cells (5). Interestingly, like the persistence of true exhausted CD8 T cells during chronic viral infection, the persistence of “exhausted-like” D^b^-NP_366−374_ T_RM_ cells is also dependent on the continuous presence of pMHC-I and co-stimulatory signaling as the induced depletion of MHC-I or the late blockade of CD28 diminished D^b^-NP_366−374_ T_RM_ cell magnitude (5). How these antigenic signals in the lung work in concert with the main cytokine (TGF-β) responsible for T_RM_ cell differentiation across a breadth of tissues is unclear.

TGF-β is an integrin-activated cytokine with widely varying effects on white blood cells from the hematopoietic stem cell (HSC) stage through to terminal differentiation (51). TGF-β mediates the fine line between immune-tolerance and appropriate activation of both the innate and adaptive immune systems (52–58). As with most of its cell-type dependent functions, effects of TGF-β on CD8 T cells can be stimulatory or inhibitory, depending on the state of differentiation (57, 59). TGF-β can raise the threshold of TCR-induced activation on naïve CD8 T cells, whereas it can induce either T_CM_-like or T_RM_-like differentiation in recently activated CD8 T cells (57, 60–62). TGF-β mediates T_RM_ cell differentiation by imparting a partially shared transcriptional footprint across a breadth of organs, however, it is the tissues themselves that govern the uniqueness of the footprint such as what metabolites T_RM_ cells use to persist (61, 63, 64). Similar to most peripheral sites, TGF-β is essential for differentiation of pulmonary T_RM_ cells of numerous antigen specificities (5, 41, 65). Interestingly, low affinity TCR-pMHC interactions leave CD8 T cells more susceptible to TGF-βR signaling which could explain their proclivity toward T_RM_ cell differentiation (47, 49). For respiratory viral infections, the effects of TGF-β signaling on T_RM_ cell generation is Smad4-independent, which may suggest non-canonical TGF-β R signaling pathways are vital for pulmonary T_RM_ cell differentiation (65, 66). Thus, it is likely the context and tissue dependent circumstances of T cell activation may govern how TGF-β contributes to T_RM_ cell heterogeneity.

### Pulmonary T_RM_ Cells Balance Immune Protection and Local Pathology

As mentioned previously, a subset of influenza-specific T_RM_ cells display an exhausted-like phenotype including high expression of PD-1. When PD-L1-PD-1 signaling in influenza infected mice is blocked at the memory stage, the magnitude of the D^b^-NP_366−374_, but not D^b^-PA_224−233_, T_RM_ cell responses was augmented (5). Furthermore, late PD-L1 blockade increases effector cytokine, particularly TNF, production by D^b^-NP_366−374_ T_RM_ cells, indicating targeting the checkpoint molecule PD-1 “rejuvenates” the exhausted-like T_RM_ cells following influenza infection. Consequently, T_RM_ cell-mediated protective immunity was enhanced upon secondary heterologous viral challenge (5). Unexpectedly, pulmonary inflammation and fibrosis were drastically exacerbated following PD-L1 blockade in a CD8 T cell-dependent manner. It is possible that enhanced production of effector molecules from an increased number of CD8 T_RM_, mediates diffuse alveolar damage in the absence of molecular regulation such as PD-1 signaling (67–69) (Figure 1). Failure to acutely repair this CD8-dependent airway damage, could result in exacerbated collagen deposition or impaired degradation suggesting macrophage and/or fibroblast involvement (5, 6). These results suggest that there is a fine balance on T_RM_ cell-mediated protective immunity and lung pathology following viral pneumonia. These data also indicate that the gradual T_RM_ cell loss in the respiratory tract is perhaps a host-protective mechanism to avoid potential collateral damage to a vital organ. There are also examples of CD8 T_RM_ cells causing pathology in the skin and intestine when homeostatic controls are lost and diseases like vitiligo, psoriasis, or celiac may emerge following destruction of melanocytes, epidermal or mucosal barrier tissues, respectively (70–73). Collectively, these data indicate that one's immune-status is an important regulator of the potential harm to local tissue brought on by unruly T_RM_ cell activation.

### Altered Immune Homeostasis in Advanced Age

Many hurdles exist with regards to provoking efficacious adaptive immune responses in those of advanced age (>70 years)—the demographic that may benefit most from vaccines for emerging pathogens. To understand how immune responses in aged and young hosts proceed differently, we need to understand how the innate and adaptive systems differ globally during the natural aging process. Low-grade systemic inflammation under homeostatic conditions is a hallmark signature of aging, but to what degree it impairs protective immune responses is unclear. This so-called “inflamm-aging” may in-part, be mediated by enhanced myelopoiesis during aging, another hallmark of aging (74). Interestingly plasma cell accumulation in the bone marrow has been shown to drive the myeloid bias with age. Plasma cells remodel bone marrow stroma that govern hematopoiesis, *via* provision of tumor necrosis factor (TNF), a principle “inflamm-aging” cytokine (75). The skewing of hematopoietic output leads to an age-related decline of naive lymphocytes in the circulation (74–76). Aside from decreased B cell numbers, there is a wide range of age-related functional changes in peripheral B cells that could affect antibody responses to vaccines in the elderly (77–79). Bone marrow is not the only primary lymphoid tissue that suffers age-related output predicaments that might influence vaccine efficacy in the elderly.

Thymic involution starts in the earliest years of life and drops output of naive T cells ~10-fold past the age of 40 (80). This impacts the circulatory T cell compartment as there are fewer recent thymic emigrants seeding secondary lymphoid tissue. For unknown reasons, this affects the diversity of the naïve CD8 compartment more than the CD4 T cell compartment (81). Thus, with age, CD8 memory T cells are enriched and TCR repertoires are likely narrowed across tissues (80–85). Notably, if memory CD8 T cells are formed early in life, they likely provide life-long diverse secondary responses (86, 87).

However, the ability to generate new memory is dependent on naive CD8 T cells, which in our later years (mouse and human), skew to a more differentiated state with the majority exhibiting immuno-senescence, characterized by high signaling thresholds for activation and proliferation (88–91). Moreover, once lymphocytes exit their developmental sites and emigrate to secondary lymph tissue, they encounter age-related stromal deterioration influencing their organization within lymph nodes (92). The above confounders likely affect naïve lymphocyte generation, maintenance, activation and in sum, negatively impact formation of protective immunity toward pathogens and vaccines (85, 93).

### The Aged Environment Provokes Malfunctional CD8 T_RM_ Cell Accumulation

**Figure 2:**
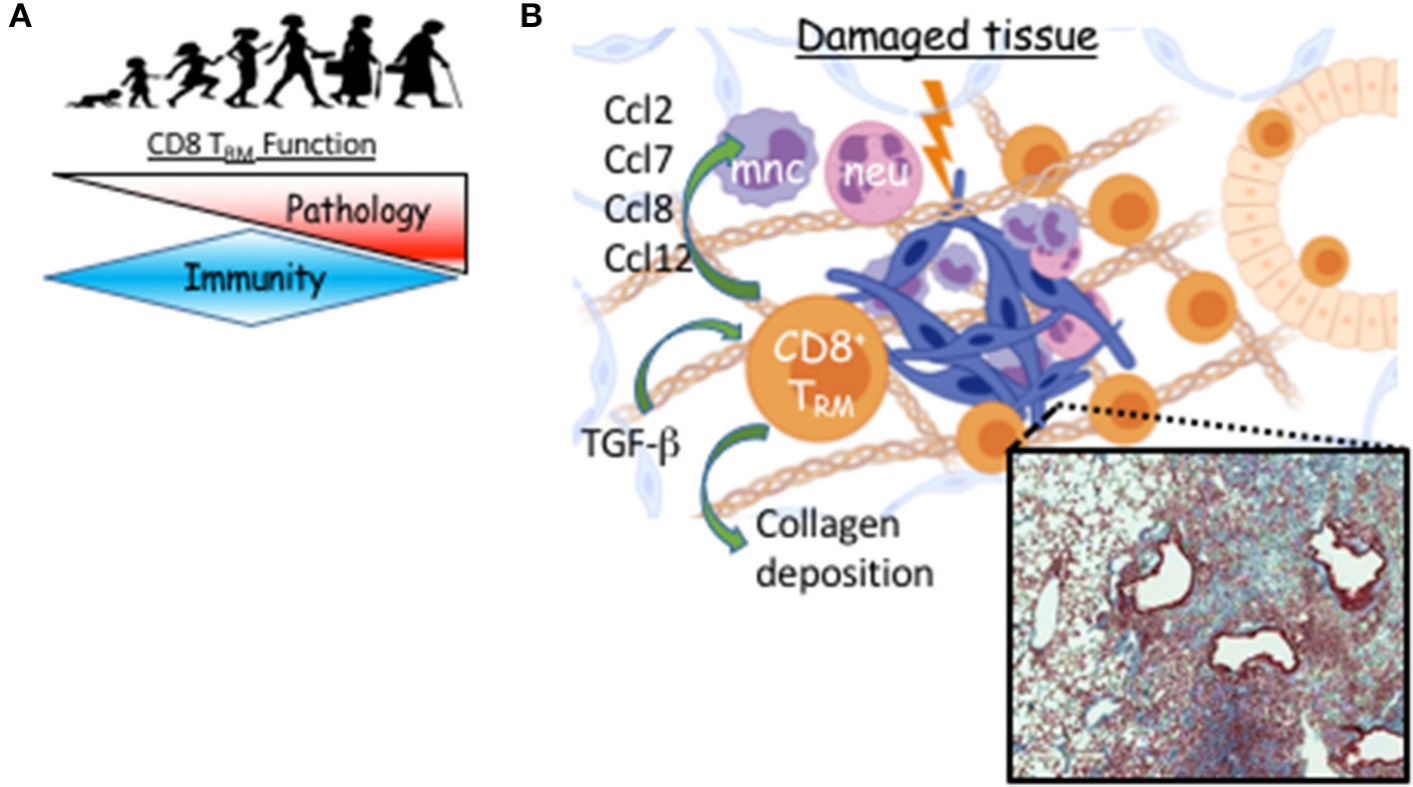
T_RM_ cell-mediated long-term sequelae post viral pneumonia during aging. **(A)** T_RM_ function switches from immune protection to pathology as we age. **(B)** Following viral pneumonia, CD8 T_RM_ cells accumulate in aged lungs where their differentiation is TGF-b–dependent. Instead of providing increased immune protection, they provoke pathology, likely through direct or indirect recruitment of myeloid cells that contribute to unresolved inflammation and prevention of collagen degradation. Micrograph is Masson's trichrome stained lung from aged mouse 60 days post-H1N1 infection. Blue is digitally enhanced collagen deposition which is dependent on CD8 T_RM_ cells (6). Created with BioRender.

One of the first clinical observations in the current pandemic was that mortality and severe morbidity in COVID-19 disproportionately affects those of advanced age (94). This is also true of most severe influenza seasons (95). Severe influenza-like illness are associated with delayed, but prolonged innate and adaptive responses during the effector phase (96). We have recently examined pulmonary CD8 T_RM_ cell responses in young (2 months) and aged (20–22 months) C57BL/6 mice following influenza infection. Aging is associated with the decreased potential of circulating memory T cell generation (97). In sharp contrast, lungs from aged mice have 40-fold more CD8 T_RM_ cells compared to those of young lungs (6). Transfer of CD8 T cells from young mice into the aged hosts results in increased accumulation of memory T cells derived from young mice in the aged lungs following influenza infection. This indicates that the aged environment provokes exaggerated accumulation of T_RM_ cells (6). We found higher levels of *Tgfb*1 transcript in the aged lungs and the accumulation of T_RM_ cells in aged hosts was largely TGF-β dependent (Figure 2). Relatedly, Chikungunya virus infection in aged mice leads to heightened and dysregulated TGF-β production that exacerbates pathology (98).

Of note, alveolar macrophage numbers and function dwindle with age (99). Given the suppressive roles of alveolar macrophages in T_RM_ cell generation (38), it could be possible that diminished alveolar macrophage function may aid the exaggerated development of T_RM_ cells during aging. Notably, many factors change in the aged lung that have not been investigated in the context of T_RM_ accumulation. DAVID analysis of the aged lung transcriptome indicates decreased cell cycle with increased extracellular matrix and cell adhesion gene programs (100). Human Lung Cell Atlas (HLCA) data indicates these changes are accompanied by increases in fibroblasts and neuroendocrine populations and a drop in Type II pneumocytes (100, 101). Additionally, the stroma may be more apt to prompt inflammation in lungs of aged individuals (102). Nevertheless, the data indicate that the aged environment enhances T_RM_ cell accumulation after a single *de novo* response, suggesting that the aged lung is fertile ground for T_RM_ cell differentiation. In contrast, there is a reduced generation of lung T_RM_ cells following influenza infection in infant mice, largely due to T cell-intrinsic defects (103).

Our data suggest that memory T cells can robustly accumulate in mucosal tissue during aging following a single round of viral challenge. Yet, aged individuals still have impaired protective responses following vaccines or respiratory viral infections which has been attributed to memory CD8 T cell function (104). To resolve the discrepancy, we performed single cell (sc) RNA-seq on young or aged T_RM_ cells against the major influenza protective epitope D^b^-NP_366−374_. Our results found that T_RM_ cells isolated from aged lungs lack a subpopulation characterized by high expression of molecules involved in TCR signaling and effector function (6). Consequently, we found that aged mice exhibit impaired T_RM_ cell-mediated protective immunity against heterologous viral rechallenge compared to those of young mice. Thus, aging facilitates the accumulation of dysfunctional T_RM_ cells in the respiratory tract, which explains the phenomena that aged individuals have increased susceptibility of influenza-associated severe diseases despite the robust presence of influenza-specific T_RM_ cells in the respiratory tract. Given the current spread of SARS-CoV2 infection among the elderly population, it would be important to determine whether SARS-CoV2-specific T_RM_ cells exhibit similar functional impairment during aging as the T_RM_ cell-mediated protection would be a key determinant of respiratory immunity during secondary exposure to the virus.

If these newly formed T_RM_ cells are not providing protection, what is their role in the tissue during aging? To address the question, we depleted either circulating, or circulating plus resident CD8 T cells and examined the long-term effects on organ-level transcription and histopathology (6). Depletion of the resident CD8 T cells that were not providing protection against subsequent influenza infection, led to resolution of pulmonary inflammation in aged hosts while concomitantly decreasing the inflammatory environment at the transcriptional level, particularly, chemokines involved in recruiting monocytes and neutrophils (Figure 2) (6). Further, long-term age-related infection-induced exacerbation of collagen deposition was mitigated in the absence of parenchymal CD8 T cells (Figure 2). Establishment of pulmonary T_RM_ in IAV infection models depends on local presentation of antigen, likely *via* monocyte-derived macrophages and/or dendritic cells, which we find sustained in the aged lung parenchyma (40, 46, 105). Infiltrating monocyte-derived macrophages have been shown to exacerbate collagen-deposition following influenza infection (106). Collectively, this could indicate the aged environment provokes accumulation of pulmonary T_RM_ cells that support ongoing inflammation of the organ contributing to its poor repair following respiratory viral pneumonia.

As discussed above, SARS-CoV2 infection disproportionately affects aged individuals. Of particular relevance is the observation of severe COVID-19 patients presenting both with CD8 T cell lymphopenia in the blood, but large number of T_RM_-like CD8 T cells in the airways (107). Notably, emerging evidence has suggested that a large proportion of COVID-19 patients exhibit pulmonary and extrapulmonary symptoms 6 months after recovery from the acute morbidity (108). Particularly, it is predicted that a large number of severe COVID-19 patients will develop persistent lung damage and fibrosis as observed in patients infected with SARS-CoV and MERS (109–113). Notably, TGF-β activating integrin is upregulated in fibrotic lung lesions in COVID-19 patients 2 months post-infection, which could support fibrosis and T_RM_ cell maintenance (114). It would be critically significant to examine whether malfunctional CD8 T_RM_ cells contribute to the long-term fibrotic sequelae of SARS-CoV2 infection.

While viral-specific pathogenic CD8 T cells have not been found in human tissue to-date, plausible candidates may now be on the radar. Age-associated granzyme K-expressing CD8 T cells are enriched in the T effector memory compartment in human blood (81). Age-associated CD8 T cell counterparts in mice were identified by expression of the effector molecule granzyme K, the checkpoint molecule PD-1, integrin CD49d, and the transcription factor TOX and are enriched in blood and across tissues (spleen, peritoneum, lungs, liver, and white adipose tissue) with age. The aged environment conferred this phenotype to young CD8 T cells in adoptive transfer models. While the TCR repertoires of age-associated CD8 T cells were clonally narrowed within each host across tissues, between hosts, their TCR sequences were diverse, suggesting either microbial-specific or stochastic differentiation. It is important to note that these age-associated CD8 T cells are transcriptionally distinct from senescent virtual memory CD8 T cells also enriched with age (88). It's unclear how granzyme K^+^ age-associated CD8 T cells behave in an immune response. While their phenotype (PD-1^Hi^ TOX^+^) is typically associated with CD8 T cell exhaustion, recombinant granzyme K augmented cytokine and chemokine production from senescent fibroblasts *in vitro* (81). Activation of local age-associated CD8 T cells may thus provoke inflammation and potentially influence tissue remodeling and senescence associated secretion phenotypes.

### Age-Related Pulmonary Fibrosis

Examples of age-related increases in lung tissue disrepair abound and are found commonly in idiopathic pulmonary fibrosis (IPF) (115, 116). IPF is an interstitial pneumonic disease that results in alveoli involved in gas exchange being progressively replaced by scar tissue with a 20% 5 year survivability (117). No treatment can reverse the process once started. As its namesake would suggest, IPF has no known single cause and it is unclear how the tissue becomes damaged and fails to repair. It is notable that IPF shares some features of viral pneumonia sequelae including COVID-19, most prominent of which is collagen accumulation which can lead to fibrosis (118). We described an increased number of CD8 T cells in the parenchyma surrounding lesions in IPF patients (5). It is plausible that these patients lost the battle for homeostatic control of local memory T cells that can mediate bystander inflammation. Of note, respiratory T cells have a role in dysfunctional wound repair resulting in fibrosis in acute lung injury models (119). Further, one of the frontline treatments (Nintedanib) that slows development of IPF by presumably targeting the kinase activities of PDGF, FGF, and VEGF receptors, inhibits src family tyrosine kinases, including the crucial T cell activating kinase Lck, with similar IC_50_ values (120, 121). This could implicate dampened T cell activity as a partial mechanism slowing fibrotic progression in the lung. Thus, while lung damage and repair models can happen in lymphocyte-scarce environments, certain T cell subsets exacerbate fibrosis and the jury may need to be recalled as to whether local T cells play a role in IPF pathogenesis and potentially viral pneumonic sequelae in humans.

## Conclusions

Although pulmonary resident memory CD8 T cells have shown outstanding immune-protective capacity, this does not seem to be the case in aged hosts following respiratory viral infections. In contrast, resident CD8 T cells mediate pathology during the disease course leading to non-resolution of lung inflammation in aged hosts. Unexpectedly, aged hosts accumulate local T_RM_ cells despite a poor response in the circulation (6). This suggests efforts should be retooled to restore their protective immunity (122) and mitigate their pathogenic capacity rather than recruit more to the mucosa. These opposing features of T_RM_ cells in young and aged hosts may identify a balance between immune protection and pathology and shed light on their teleological existence in a vital organ. While recent work has highlighted the cellular and molecular networks that mediate pulmonary T_RM_ density in young healthy hosts, we are just beginning to understand the potential they have to mediate damage when homeostatic controls are lost, e.g. through the aging process. Understanding the mechanisms modulating the balance of T_RM_ cell-mediated immunity vs. pathogenicity will be important to selectively harness the beneficial function of T_RM_ cells and simultaneously mitigate their pathogenic potential.

## Author Contributions

NG and JS wrote and IC was responsible for editing the manuscript. All authors contributed to the article and approved the submitted version.

## Conflict of Interest

The authors declare that the research was conducted in the absence of any commercial or financial relationships that could be construed as a potential conflict of interest.
